# Immunoregulation by antigen-presenting cells in human intestinal lamina propria

**DOI:** 10.3389/fimmu.2023.1138971

**Published:** 2023-02-09

**Authors:** Takayuki Ogino, Kiyoshi Takeda

**Affiliations:** ^1^ Department of Gastroenterological Surgery, Graduate School of Medicine, Osaka University, Suita, Japan; ^2^ Department of Therapeutics for Inflammatory Bowel Diseases, Graduate School of Medicine, Osaka University, Suita, Japan; ^3^ Laboratory of Immune Regulation, Department of Microbiology and Immunology, Graduate School of Medicine, Osaka University, Suita, Japan; ^4^ Immunology Frontier Research Center, Osaka University, Suita, Japan

**Keywords:** inflammatory bowel disease, antigen-presenting cell, mucosal immunology, macrophage, dendritic cell, helper T cell

## Abstract

Antigen-presenting cells, including macrophages and dendritic cells, are a type of innate immune cells that can induce the differentiation of T cells and activate the adaptive immune response. In recent years, diverse subsets of macrophages and dendritic cells have been identified in the intestinal lamina propria of mice and humans. These subsets contribute to the maintenance of intestinal tissue homeostasis by regulating the adaptive immune system and epithelial barrier function through interaction with intestinal bacteria. Further investigation of the roles of antigen-presenting cells localized in the intestinal tract may lead to the elucidation of inflammatory bowel disease pathology and the development of novel treatment approaches.

## 1 Introduction

The intestinal tract is constantly exposed to foreign substances, such as microorganisms and dietary antigens. The microorganisms, including intestinal bacteria, viruses, and fungi, form a diverse ecosystem in the intestine. The intestinal immune system stimulates an inflammatory response as a biological defense against pathogens and induces immune tolerance towards the indigenous intestinal bacterial flora and dietary antigens. Therefore, a balance in the intestinal immune system is crucial for maintaining tissue homeostasis.

Antigen-presenting cells, including macrophages and dendritic cells, are a type of innate immune cells. These cells present the acquired antigens to helper T cells (Th) and activate the adaptive immune response by inducing the differentiation of naive T cells into effector T cells (1, 2). Intestinal macrophages induce an immediate inflammatory reaction upon detecting the entry of pathogenic microorganisms in the intestine. However, because constitutive activation of macrophages and dendritic cells leads to an imbalance in the adaptive immune system, intestinal macrophages, which are constantly exposed to antigens, must maintain low responsiveness in the steady state. Intestinal macrophages have a high phagocytic capacity, produce higher levels of anti-inflammatory cytokine IL-10 than spleen macrophages, induce differentiation of regulatory T cells (Treg), and regulate inflammatory response by Th1/Th17 (3–6). Administration of antibodies against TNF-α, IL-12/23p40, and IL-6 produced by innate immune cells is effective for treating inflammatory bowel disease (IBD) (7, 8). Thus, the regulation of the activation of the adaptive immune system by innate immune cells may be necessary for maintaining the homeostasis of intestinal tissue. Diverse populations of innate immune cells have been identified in the intestinal lamina propria of mice, with each cell population contributing to the maintenance of intestinal tissue homeostasis by regulating the adaptive immune system and epithelial barrier function through interaction with intestinal bacteria (9–13).

A rapid increase in the incidence of IBD, which is broadly divided into Crohn’s disease (CD) and ulcerative colitis (UC), has been observed in Asia owing to the westernization of diet and lifestyle (14). IBD is an intractable disease in which inflammation in intestinal tissue recurs chronically (15, 16); however, many aspects of its detailed pathogenic mechanism remain unclear. The adaptive immune system, including Th1/Th2 imbalance and excessive Th17-induced immunity, has been the focus of discussion in the pathology of IBD. Furthermore, a counterpart of the mouse innate immune cell population has recently been identified in the human intestinal tract, and we have begun to understand that abnormal activation of innate immune cells is greatly involved in the onset of IBD by inducing abnormalities in the adaptive immune system (17–19).

**Figure 1:**
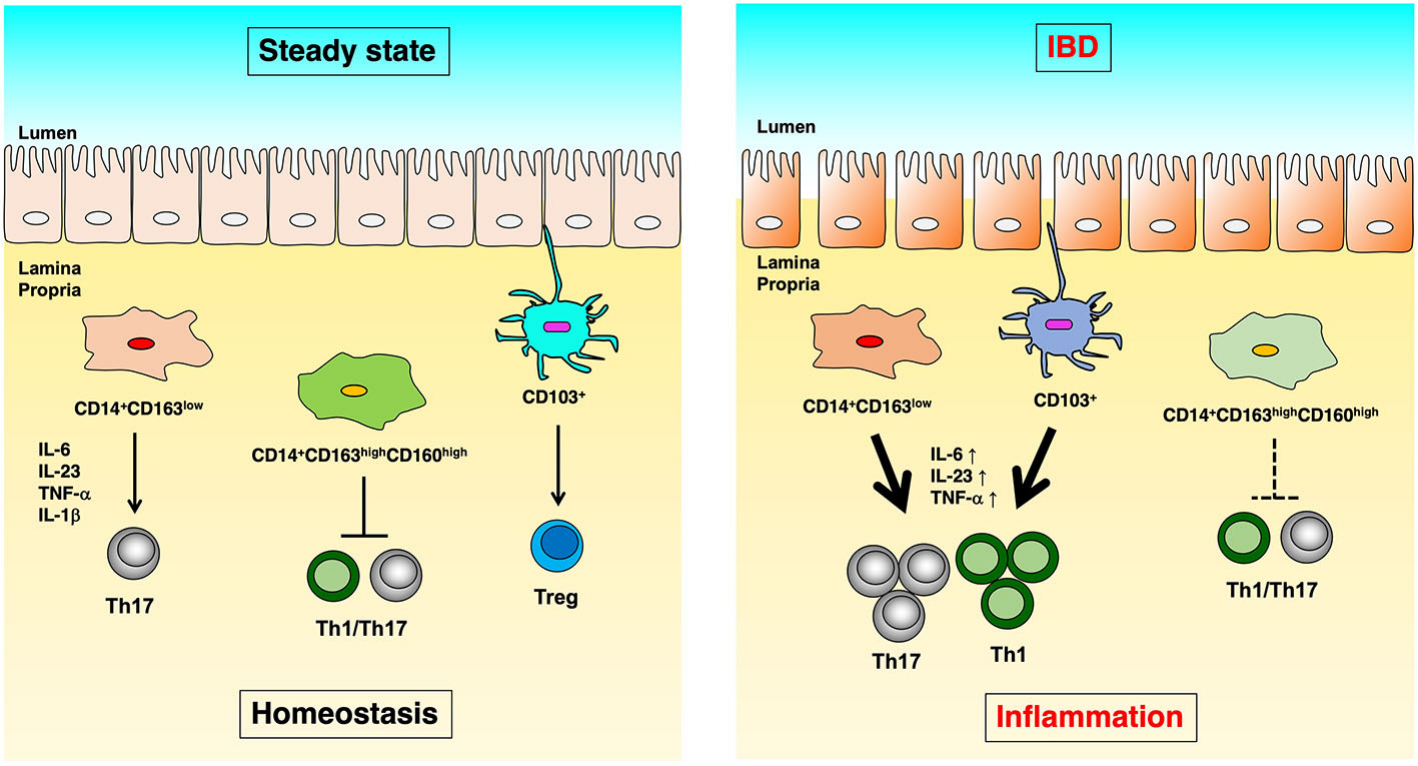
The difference of immunoregulation between steady state and IBD. In human intestinal lamina propria, CD14+CD163low cells induce Th17 differentiation in an IL-6, IL-23p19, TNF-α, and IL-1β dependent manner. CD14+CD163highCD160high cells suppress the proliferation of CD4+ T cells. CD103+ dendritic cells induce Treg cells in steady-state (left). On the other hand, in Crohn’s disease, CD14+CD163low cells have significantly increased expression of IL-6, IL-23p19, and TNF-α, as well as enhanced Th17 induction capacity. In ulcerative colitis, CD14+CD163highCD160high cells reduce the ability to suppress CD4+ T cell proliferation, and CD103+ dendritic cells showed reduced Treg- and enhanced Th17-inducing capacity (right).

We identified CD14+CD163low cells with Th17 inducibility (20), CD14+CD163highCD160high cells with anti-inflammatory function (21), and CD14-CD103 cells with Treg induction capacity as populations of innate immune cells in the human intestinal tract (22), and we revealed their functional abnormalities in IBD patients (**Figure 1**). In this article, we focused on antigen-presenting cells localized in the human intestinal tract and introduced the mechanisms involved in the maintenance of intestinal homeostasis and the pathology of IBD.

## 2 Genome-wide association study of IBD

NOD2, which is a pattern recognition receptor (PRR), and ATG16Ll and IRGM, which are autophagy-related factors, were identified as CD-related genes *via* genome association analysis using single nucleotide polymorphisms (23–25). NOD2 is one of the PRRs that induce inflammatory reactions through bacterial recognition, and there have been many discussions on the mechanism by which NOD2 gene mutations are involved in the onset of CD (26). It has been reported that NOD signaling induces the production of IL-10 from macrophages and that the expression of NOD2 in intestinal macrophages suppresses the activation of NF-κB and the TLR2-dependent production of Thl-inducing cytokines, regulating intestinal inflammation (27–29).

In addition, IL-23R, IL-12/23p40, STAT3, and IL-10, which are involved in the innate immune response, have been identified as common susceptibility genes for CD and UC (30, 31), suggesting the regulation of the adaptive immune system by appropriate innate immune response functions to suppress the onset of IBD.

## 3 CD14+CD163low cells with Th17 induction capacity

Human intestinal macrophages localized in the intestinal lamina propria of healthy individuals are divided into CD14-CD33+ and CD14+ macrophages. CD14-CD33+ macrophages have high phagocytic and bactericidal capacities but have low responsiveness to TLR (Toll-like receptor) ligand stimulation and low inflammatory cytokine production capacity (32, 33).

Compared to healthy individuals, CD patients have an increased number of CD14+ macrophages, and their intestinal bacteria-dependent production of inflammatory cytokines is enhanced. It has been found that excessive IL-23 production by CD14+ macrophages triggers Th1/Th17 responses involved in inflammation-associated tissue destruction and that IFN-γ produced by Th1 cells induces abnormal differentiation of IL-23-producing macrophages, causing the persistence of the inflammatory response (20, 33, 34).

CD14+ macrophages in the human intestinal lamina propria are further divided into CD163low and CD163high cells. CD14+CD163low cells express TLR2, TLR4, and TLR5 and produce high levels of inflammatory cytokines, including IL-6, IL-23p19, TNF-α, and IL-1β, whereas CD163high cells express TLR5 and produce high levels of IL-10. CD163low cells induce Th17 differentiation in an IL-6-, IL-23p19-, TNF-α-, and IL-1β-dependent manner, and CD163low cells from CD patients have significantly increased expression of IL-6, IL-23p19, and TNF-α, as well as enhanced Th17 induction capacity. These findings suggest the involvement of abnormal activation of CD14+CD163low cells in the onset and pathology of CD (20).

## 4 CD14+CD163highCD160high cells with T cell-proliferation suppression capacity

Compared to CD14+CD163low cells that induce Th17, CD14+CD163high cells localized in the lamina propria of the large intestine in healthy individuals show a high phagocytic capacity and produce high levels of IL-10. IL-10 gene-deficient mice (IL10 -/- mice) and macrophage-specific Stat3-deficient mice (LysM-cre; Stat3 f/- mice) spontaneously develop enteritis (35–37). IκBNS, an IL-10-inducible molecule, binds to NF-κB p50 in intestinal macrophages to suppress the production of inflammatory cytokines and negatively regulate the differentiation of Th1/Th17 cells (38, 39). CX3CR1high cells of the mouse large intestine produce high levels of IL-10, express high levels of cell adhesion molecules ICAM-1/VCAM-1, and preferentially contact with CD4+ T cells (40). However, since they suppress the expression of CD80/CD86 in an IL-10/Stat3 signaling-dependent manner, it has been reported that CX3CR1high cells of the mouse large intestine do not induce proliferation of the contacting CD4+ T cells and suppress Th1/Th17-dependent intestinal inflammation (39). In addition, while TLR signaling is also suppressed *via* activation of IL-10/Stat3 signaling, activation of TLR signaling by intestinal bacteria restricts migration of CX3CR1high cells to MLN (41, 42).

We investigated the possibility that human intestinal CD14+CD163high cells may contain the counterpart of mouse CX3CR1high cells. Through the examination of various surface markers, we further subdivided CD14+CD163high cells into CD160low and CD160high cells. CD14+CD163highCD160high cells showed a gene expression pattern highly similar to that of CX3CRlhigh cells of the mouse large intestine, expressing high levels of VCAM-1 and reduced levels of CD80/CD86. CD14+CD163highCD160high cells did not induce differentiation of effector T cells and suppressed the proliferation of CD4+ T cells in a Treg cell-independent manner, suggesting that CD14+CD163highCD160high cells are anti-inflammatory macrophages that are the counterpart of mouse intestinal CX3CR1 high cells. The lamina propria of the large intestine in UC patients showed a markedly decreased number of CD14+CD163highCD160high cells, decreased suppression capacity of CD4+ T cell proliferation, and elevated expression of CD80/CD86 (21). These findings suggest that the maintenance of homeostasis by CD14+CD163highCDl60high cells in the intestinal lamina propria is important for suppressing inflammation in UC patients.

## 5 CD103+ dendritic cells with Treg induction capacity

Treg cells regulate the activation of effector T cells, and peripheral Treg cells are induced from naive T cells in the presence of TGF-β, IL-2, and retinoic acid (5, 6, 43). It has been reported that transferring CD4+CD25+Foxp3+ Treg cells into chronic colitis model mice suppresses the onset of colitis (44), indicating the importance of Treg cells in the pathology of IBD.

HLA-DRhigh CD103+ cells localized in the lamina propria of the human large intestine are dendritic cells, and they have been reported to induce Treg (45, 46). CD 103+ dendritic cells localized in mesenteric lymph nodes induce CD8+ T cells to express the intestinal homing receptor CCR9 and integrin α4β7 in a retinoic acid-dependent manner (47). Furthermore, intestinal epithelial cells promote the differentiation of CD103+ dendritic cells (48). It has been found that although CD103+ dendritic cells in UC patients have a markedly reduced Treg cell induction capacity, they show an increased expression of the inflammatory cytokines IL-23a, IL-6, and TNF-α, as well as enhanced Th17-inducing capacity (22). These findings suggest the involvement of functional abnormalities of CD103+ dendritic cells in the pathology of UC patients.

## 6 Development of treatment targeting macrophages

As mentioned above, several autophagy-related genes have been identified as CD susceptibility genes, and the dysfunction of autophagy is involved in IBD etiology. We screened a library containing 3,922 natural extracts for autophagy-activating factors using a high-throughput assay system. Among the extracts identified as autophagy-activating factors, *Sanguisorba officinalis* L. (SO) showed an effect on DSS-induced colitis in mice. To clarify the mechanism of SO, epithelial cell and macrophage-specific Atg7-deficient mice (Villin-cre and LysM-cre; Atg7 f/f mice) were evaluated. SO-mediated amelioration of DSS-induced colitis was observed in Villin-cre; Atg7 f/f mice. However, SO did not have an effect on LysM-cre; Atg7 f/f mice (49). The lack of autophagy in macrophages reportedly results in reduced polarization of anti-inflammatory macrophages in liver and fat tissue (50, 51). In large intestinal CD11b+ cells, SO decreased the expression of IL-6 and IL-23a, and enhanced expression of IL-10 and marker genes of anti-inflammatory macrophages, such as Relma, CD206, and Arg1. During DSS-induced colitis, SO did not affect the differentiation of CX3CR1highCD11b+ macrophages, which have anti-inflammatory properties (41). These findings indicate that induction of autophagy leads to the amelioration of colitis by providing anti-inflammatory profiles to intestinal macrophages. In IBD patients, intestinal CD14+ macrophages show facilitated the production of IL-6 and IL-23 in response to commensal bacteria (20, 33, 34). Thus, it would be important to examine whether SO provides anti-inflammatory profiles in human intestinal macrophages for the development of novel therapeutic interventions for IBD.

Both dendritic cells and macrophages can be induced from human iPS (induced pluripotent stem) cells *via* monocytes. Application research on iPS cell-derived antigen-presenting cells is in progress in fields that include disease model construction, antitumor therapy development, and infection control. The differentiation induction method of iPS cell-derived antigen-presenting cells can be divided into two parts: differentiation from iPS cells to monocytes *via* blood cell progenitor cells and differentiation from monocytes to dendritic cells and macrophages. Monocytic cells can be induced by adding M-CSF (macrophage colony stimulating factor) and GM-CSF (granulocyte macrophage colony stimulating factor) to iPS cells. Stimulation of monocytes with GM-CSF and IL-4 induces dendritic cells, while their stimulation with M-CSF induces macrophages. The induced dendritic cells can promote the proliferation of T cells, and the macrophages exhibit phenotypes according to each macrophage subtype in response to polarization signals to M1/M2 macrophages (52–54).

Problems in the clinical application of antigen presenting cells include the limited number of cells that can be collected from human specimens and the prolonged time required for culturing from iPS cells, which yields a limited number of cells. Culture systems for antigen-presenting cells are also being developed, but they are still at an early stage of development (55). In addition, immune therapy using antigen-presenting cells must consider serious adverse effects caused by autoimmune reactions (56). However, the use of antigen-presenting cells in combination with other immune therapies may have profound therapeutic effects, and future development of the field is anticipated.

## 7 Conclusion

Human intestinal lamina propria contains CD14+CD163low and CD14+CD163high macrophages as well as a wide variety of innate immune cells, such as CD103+ dendritic cells. Each cell population plays an important role in the maintenance of intestinal tissue homeostasis by regulating the adaptive immune system through different mechanisms. We have begun to understand that the reduction and dysfunction of these antigen-presenting cells are deeply involved in the onset of IBD. In the clinical application of antigen-presenting cells, the use of cells collected from human surgical specimens and iPS-derived cells is expected to lead to the elucidation of IBD etiology and the development of a novel treatment.

## Author contributions

TO wrote the manuscript. TO and KT finalized the manuscript. All authors contributed to the article and approved the submitted version.
